# Meal Replacements for Weight-Related Complications in Type 2 Diabetes: What Is the State of the Evidence?

**DOI:** 10.3389/fendo.2022.875535

**Published:** 2022-07-28

**Authors:** Jarvis C. Noronha, Cyril WC. Kendall, John L. Sievenpiper

**Affiliations:** ^1^ Toronto 3D (Diet, Digestive Tract and Disease) Knowledge Synthesis and Clinical Trials Unit, Clinical Nutrition and Risk Factor Modification Centre, St. Michael’s Hospital, Toronto, ON, Canada; ^2^ School of Medicine, Faculty of Medicine, The University of Queensland, Brisbane, QLD, Australia; ^3^ College of Pharmacy and Nutrition, University of Saskatchewan, Saskatoon, SK, Canada; ^4^ Department of Nutritional Sciences, Temerty Faculty of Medicine, University of Toronto, Toronto, ON, Canada; ^5^ Department of Medicine, Temerty Faculty of Medicine, University of Toronto, Toronto, ON, Canada; ^6^ Division of Endocrinology and Metabolism, St. Michael’s Hospital Toronto, Toronto, ON, Canada; ^7^ Li Ka Shing Knowledge Institute, St. Michael’s Hospital Toronto, Toronto, ON, Canada

**Keywords:** meal replacement, lifestyle intervention program, cardiometabolic risk factors, diabetes, dietary patterns

## Abstract

Comprehensive lifestyle management is a fundamental aspect of diabetes care. Clinical practice guidelines for the nutritional management of diabetes have evolved considerably over the last 25 years shifting from a focus on single nutrients to food- and dietary pattern-based recommendations. Use of meal replacements as a temporary short-term strategy to induce weight loss and then transitioning to a healthier dietary pattern (e.g., Mediterranean or Portfolio) for weight loss maintenance fits well with this new shift in focus of clinical practice guidelines. As adherence is the most important determinant for attaining the benefits of any diet, health professionals should recommend evidence-based dietary patterns (including meal replacements) that align best with the patient’s values, preferences, and treatment goals.

## Introduction

Dietary management is important in preventing diabetes and managing existing diabetes. Over the last 25 years, clinical practice guidelines for the nutritional management of diabetes have shifted from a focus on single nutrients to food- and dietary pattern-based strategies (1–5). Furthermore, there is also a wide recognition that there is no one best diet. There are several food- and dietary pattern-based approaches that have advantages and disadvantages. Thus, it is important for healthcare providers to align these advantages and disadvantages with the values, preferences, and treatment goals of the patient to obtain the greatest adherence and ultimately, the greatest benefit in diabetes prevention and management.

One dietary approach is the utilization of meal replacements to facilitate rapid weight loss followed by a transition to a healthy dietary pattern for weight loss maintenance. Meal replacements provide a combination of macro- and micronutrients and are frequently used to replace one or two main meals, or all meals (total diet replacement) each day. Meal replacements are often supplemented with fruits, vegetables, and nuts during or between meals to achieve the targeted daily caloric intake.

In this article, we will review evidence related to the effectiveness of comprehensive lifestyle interventions in diabetes prevention and management followed by a summary of some of these trials which utilized meal replacements. We will then summarize a systematic review and meta-analysis (SRMA) that examined the effect of liquid meal replacements as part of a weight loss diet on cardiometabolic risk factors in type 2 diabetes (T2D). Lastly, we will highlight two comprehensive dietary patterns, as examples, that patients can transition to for further improvements in cardiometabolic health and reduction of cardiovascular disease risk.

## Effectiveness of Comprehensive Lifestyle Interventions in Diabetes Prevention and Management

As stated in several clinical practice guidelines, one of the most important pillars for medical nutrition therapy in diabetes are lifestyle interventions using a multi-disciplinary approach targeting weight loss. In this section, we will review evidence from several landmark clinical trials that support its use.

### Diabetes Prevention Program (DPP)

In this large, multi-centre, randomized controlled study (6), 3234 patients at risk of developing T2D (defined as elevated fasting and post-load plasma glucose concentrations) were randomized to one of three groups: (1) a lifestyle intervention program targeting ≥7% weight loss and ≥150min of physical activity per week, (2) metformin (850mg twice daily) with standard lifestyle modification, or (3) placebo with standard lifestyle modification. After an average follow-up duration of 2.8 years, incidence of T2D was reduced by 58% and 31% in the lifestyle intervention and metformin groups, respectively, when compared to placebo (6). In the DPP Outcome Study (DPPOS) (7), which was a 10-year follow-up study since randomization to DPP, T2D incidence was reduced by 34% and 18% in the lifestyle intervention and metformin group, respectively, when compared with placebo (7). These findings suggest that prevention of T2D with lifestyle intervention can be achieved and can persist for at least 10 years.

### Look Action for Health in Diabetes (Look AHEAD) Study

In this 16-centre study (8) in the United States, 5145 overweight and obese patients with T2D were randomly assigned to either (1) participate in an intensive lifestyle intervention that targeted weight loss of >7% through caloric restriction and increased physical activity, or (2) receive diabetes support and education. After ~10 years of follow-up, the trial was stopped early for futility due to no difference in the composite primary endpoint of CV mortality, non-fatal MI, non-fatal stroke, or angina hospitalization between the two groups (8). However, several improvements in key secondary outcomes were observed. When compared to the control group, the intensive lifestyle intervention group demonstrated lower incidence of very-high-risk CKD after median follow-up of 8 years (9), reduced incidence of mild or greater depression symptoms after median follow-up of 9.6 years (10), reduced odds of non-alcoholic fatty liver disease (NAFLD) after 12 months (11), and 5-fold greater remission of obstructive sleep apnea (12). In an observational, *post-hoc* analysis, individuals who lost ≥10% of their body weight in the first year of the study had 21% lower risk of the primary endpoint compared with individuals with stable weight or weight gain (13). Thus, even though the intensive lifestyle intervention did not reduce the risk of cardiovascular morbidity or mortality when compared with the control program of diabetes support and education, other beneficial effects were observed and are of importance.

### Diabetes Remission Clinical Trial (DiRECT)

In this cluster-randomized trial (14) of 49 primary care practices in Scotland and the Tyneside region of England, 149 patients received the intervention (weight management program) and 149 patients were assigned to the control group (best practice care by guidelines). The intervention was divided into three phases (1): total diet replacement phase (weeks 0 to 12) where patients were asked to replace their regular diet with formula diet (825–853 kcal/day), (2) stepped food reintroduction phase which involved a stepped transition to a food-based diet (weeks 13 to 18), and (3) weight loss maintenance phase (weeks 19 to 104) with structured support. At 12 months, 46% of patients in the intervention group achieved diabetes remission compared with only 4% of patients in the control group (14). At 24 months, 36% of patients in the intervention group had diabetes remission compared with 3% in the control group (15). These findings are clinically important as it demonstrated that T2D is reversible with a professionally supported intensive weight management program.

### Prevention of Diabetes Through Lifestyle Intervention and Population Studies In Europe and Around The World (PREVIEW) Trial

This clinical trial (16) included both adults and children, and was conducted at eight intervention centres in eight countries (Denmark, Finland, the Netherlands, UK, Spain, Bulgaria, Australia, and New Zealand). In this study, the Cambridge Weight Plan was used to induce weight loss over the first 8 weeks. Participants who lost ≥8% of their initial body weight continued on to the 3-year weight maintenance phase of the study where they were randomized to one of four interventions: (1) high protein diet, high intensity physical activity, (2) high protein diet, moderate intensity physical activity, (3) moderate protein diet, high intensity physical activity, and (4) moderate protein diet, moderate intensity physical activity. Although there was no significant difference in 3-year T2D incidence between the four interventions, overall incidence of T2D was very low (3.1%), especially when compared to previous diabetes prevention studies (10.5%-15.8%) (6, 17). Taken together, the combination of rapid weight loss in the first 8 weeks with healthy eating and physical activity for weight loss maintenance was effective in reducing the risk of developing T2D.

## Role of Meal Replacements as Part of a Comprehensive Lifestyle Intervention in Diabetes Management

Out of the several landmark clinical trials mentioned in the previous section, three of them utilized meal replacements to induce weight loss (**Table 1**). In this section, we will review this body of evidence which support the use of meal replacements as part of a comprehensive lifestyle intervention in diabetes management.

**Table 1:** Summary of large-scale randomized controlled trials incorporating meal replacements as part of a comprehensive lifestyle intervention in diabetes management.

Study (reference)	Design	Subjects	Interventions	Results
Look AHEAD Study (8, 18, 19)	Multi-centre, randomized controlled trial	5145 OW/OB patients with T2D	Intensive lifestyle intervention (ILI) involved a caloric target of 1200-1800 kcal/day, use of meal replacement (MR) products (weeks 3 to 19 only), and >175 minutes of moderate-intensity physical activity per week Diabetes support and education (DSE) involved group sessions focused on diet, exercise and social support	2^0^ outcomes (ILI vs. DSE): – ↓ very-high-risk CKD incidence – ↓ mild or greater depression symptoms – ↓ odds of NAFLD – ↑ remission of obstructive sleep apnea Weight loss & 1^0^ outcome: participants who achieved >10% weight loss had 21% lower risk of the 1^0^ outcome (composite of death from cardiovascular causes, nonfatal myocardial infarction, nonfatal stroke, or hospitalization for angina) compared with participants who had stable weight or weight gain MR use: at year 1, participants in the highest quartile of MR use had greater odds of reaching the 7% and 10% weight loss goal than did participants in the lowest quartile of MR use
DiRECT (14)	Open-label, cluster randomized trial	298 OW/OB patients with T2D from 49 primary care practices	Intervention involved total diet replacement using formula diet for 3–5 months, stepped food reintroduction for 2–8 weeks, and structured support for long-term weight loss maintenance Control group involved best practice care by guidelines	Diabetes remission: 46% of participants in the intervention group achieved diabetes remission vs. 4% of participants in the control group MR use: total diet replacement using formula diet resulted in weight loss of 14.5 kg in participants that engaged with the intervention (completers) vs. 3 kg in non-completers
PREVIEW Study (16)	Randomized trial with 2x2 factorial design (two diets and two physical activity programs)	2326 adults with pre-diabetes	Participants completed an 8-week weight loss phase which involved a low-energy diet consisting of meal replacement products Those who achieved weight loss of >8% continued on to the weight maintenance phase (148 weeks) consisting of a combination of one of two diets (high or moderate protein) with one of two physical activity (high or moderate intensity) programs	1,857/2,326 (79.8%) participants achieved >8% weight loss during the 8-week weight loss phase No significant differences were observed in 3-year T2D incidence between diets, physical activity, or their combination Overall incidence of T2D was very low (3.1%), especially when compared to previous diabetes prevention studies (10.5%-15.8%)

CKD, chronic kidney disease; NAFLD, non-alcoholic fatty liver disease; OW/OB, overweight or obese; T2D, type 2 diabetes.

### Look AHEAD Study

Part of the intensive lifestyle intervention in the Look AHEAD study involved consumption of meal replacements (8). The four meal replacement options included, Slim-Fast (Slim-Fast Foods Company, Englewood, NJ), Glucerna (Ross Laboratories, Columbus, OH), OPTIFAST (Novartis Nutrition, Fremont, MI), and HMR (Health Management Resources, Boston, MA) (18). During weeks 3 to 19, participants were asked to replace breakfast and lunch with a liquid shake and one snack with a bar. For their evening meal, participants were instructed to consume conventional foods. Throughout the day, participants were asked to add fruits and vegetables to their diet until they met their daily calorie goal (participants <114 kg = 1,200–1,500 kcal/day; individuals ≥114 kg = 1,500–1,800 kcal/day). During months 7–12 of the study, participants were instructed to replace one meal and one snack a day with meal replacement shakes and bars to facilitate weight loss maintenance. When the relationship between meal replacement use and weight loss was examined after 1 year, participants in the highest quartile of meal replacement use were found to have four times greater odds of reaching both the 7% and 10% weight loss goal than did participants in the lowest quartile (19). These findings suggests that meal replacement use was critical in achieving weight loss and partly responsible for achieving improvements in some of the secondary outcomes mentioned in the previous section.

### DiRECT

The first phase of DiRECT (14) involved total diet replacement (TDR) for 12 weeks where a commercial (Cambridge Weight Plan) liquid formula diet (825-853 kcal/d; soups and shakes) was provided to replace usual foods. During the TDR phase, weight fell sharply by 14.5 kg, on average, among participants that engaged with the intervention (completers). In contrast, participants that started, but did not complete the TDR phase (non-completers) experienced an average weight loss of only 3.0 kg. These findings underscore the role of meal replacements in inducing rapid weight loss which helped set up the majority of the diabetes remission observed in the study.

### PREVIEW Trial

During the first 8 weeks of the trial (16), a low-energy diet (~810 kcal/d) was used to induce weight loss of ≥8% in 2,326 participants to qualify them for the next phase. Participants were provided sachets (soups, shakes and porridges) without charge from the Cambridge Weight Plan. Participants were instructed to consume 4 sachets per day, of which 3 sachets were to be dissolved in low-fat milk and 1 sachet in water (20). Out of the 2,326 participants that were enrolled in the study, 1,857 participants (79.8%) achieved ≥8% weight loss for entry into the weight maintenance phase. Overall, rapid weight loss through the use of meal replacements with the combination of healthy eating and physical activity for weight loss maintenance was successful in markedly reducing risk of developing T2D that was found in the study.

## Role of Liquid Meal Replacements as a Short-Term Option to Induce Weight Loss and Improve Other Cardiometabolic Risk Factors

In 2019, the Diabetes and Nutrition Study Group (DNSG) of the European Association for the Study of Diabetes (EASD) commissioned a SRMA using the Grading of Recommendations Assessment, Development and Evaluation (GRADE) approach to summarize the available evidence from randomized controlled trials assessing the effect of liquid meal replacements as part of a weight loss diet on cardiometabolic risk factors in overweight/obese individuals with T2D (21). Liquid meal replacements were defined as products that contained a mixture of macro- and micro-nutrients, in ready-to-drink form or powder formulas that require mixing, and were used to replace one or two main meals each day.

This SRMA identified nine trial comparisons involving 961 overweight/obese participants with T2D. The median follow-up duration across all trials was 24 weeks (range 12–52). In the intervention arms, median daily caloric intake was ~1500 kcal/day with ~48% calories from carbohydrates, ~30% from fat and ~20% from protein. The median dose of liquid meal replacements represented ~20% of daily energy intake per day. Liquid meal replacements included, Glucerna SR (four of nine trials), SlimFast (two of nine trials), Medifast (one of nine trials), Probiotec Formula (one of nine trials), and Microdiet (one of nine trials). The comparators in these trials were low-calorie diets using food-exchange systems (four of nine trials), self-selected foods (four of nine trials), and a diet book (one of nine trials).

**Figure 1:**
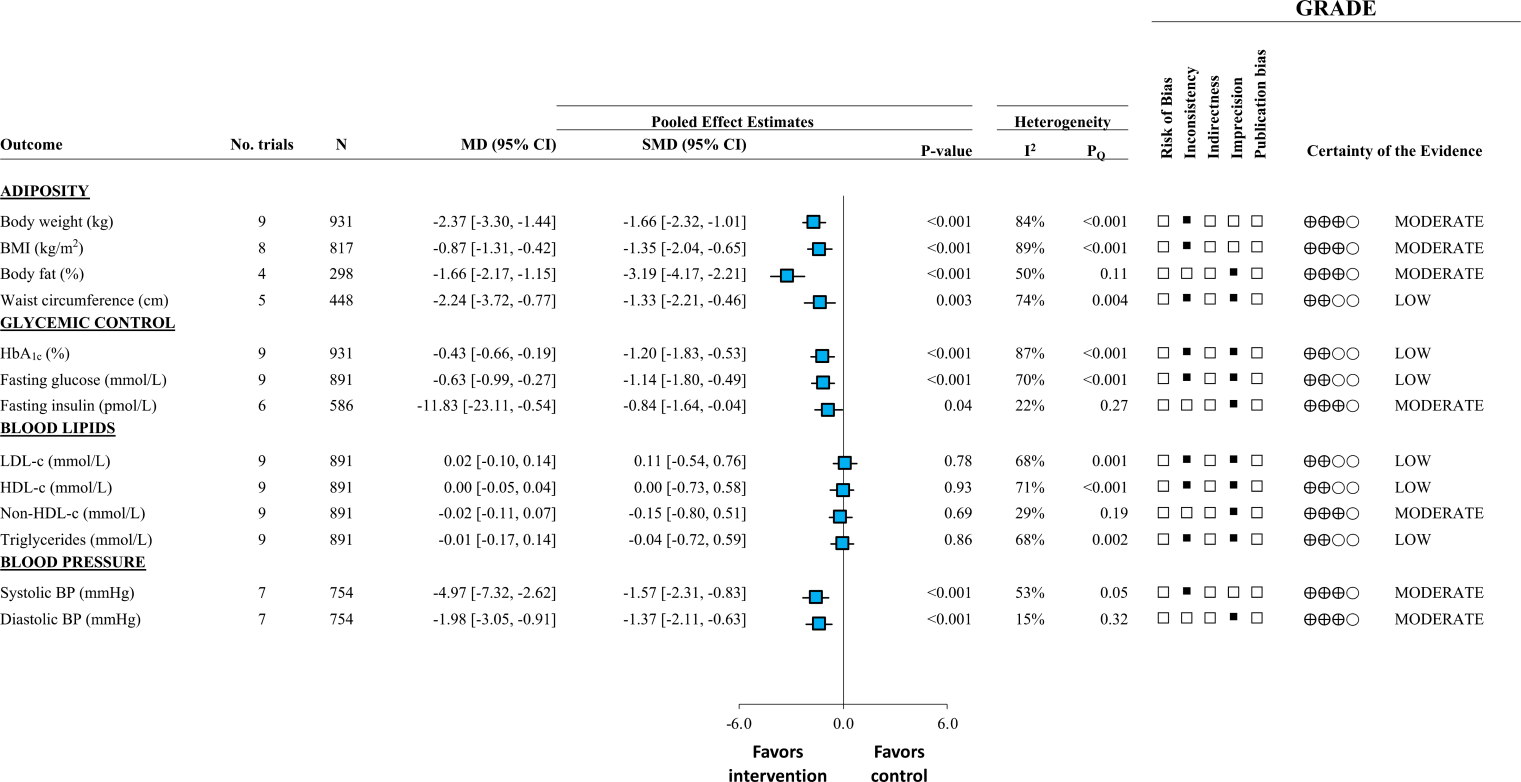
Adapted from Noronha et al., 2019 (21). Summary of pooled effect estimates with GRADE assessments of randomized controlled trials investigating the effect of liquid meal replacements as part of a weight loss diet (intervention) compared with traditional low-calorie diets (control) on cardiometabolic risk factors. Estimates are expressed as mean differences (MDs) with 95% CIs and standardized MDs (SMDs) with 95% for visualization purposes. SMDs are represented by the blue square and 95% CIs by the line through the blue square.

**Figure 1** outlines the summary of findings from this SRMA. There was moderate certainty for reductions in body weight (~2.4 kg), BMI (~1kg/m^2^), body fat (~2%), fasting insulin (~12pmol/L), and systolic blood pressure (~5 mmHg). There was low certainty for the reductions in waist circumference (~2.2 cm), HbA1c (~0.43%), and fasting glucose (~0.6 mmol/L). There was no effect on blood lipids. Removal of trials that used non-diabetes specific formulas resulted 25-30% greater reductions in the overall analysis suggesting that there may be an added benefit with diabetes-specific formulas. Overall, liquid meal replacements as part of a weight loss diet were effective as a temporary, short to moderate term (1 month to 1 year) intervention in overweight or obese individuals with T2D (21).

## Dietary Patterns for Long-Term Weight Maintenance and Additional Cardiovascular Benefits Beyond Weight Loss

From the perspective of utilizing meal replacements as a tool to temporarily induce weight loss, which dietary patterns can be used to obtain health benefits over the longer term? In this section, we review evidence for the Mediterranean and Portfolio dietary patterns as two examples of potential dietary strategies for weight loss maintenance and cardiometabolic improvement over the longer-term.

### Mediterranean Diet

The Mediterranean diet is characterized by a high consumption of fruits, vegetables, legumes, nuts, seeds, cereals and whole grains; moderate-to-high consumption of olive oil as the primary source of fat; low-to-moderate consumption of dairy products, fish and poultry; low consumption of red meat; and low-to-moderate consumption of wine, mainly during meals (3). The PREvención con DIeta MEDiterránea (PREDIMED) trial was a large randomized controlled trial (N=7447) aimed at investigating whether a Mediterranean diet with extra-virgin olive oil or nuts would reduce rates of myocardial infarction (MI), cerebral vascular accidents (CVA), or cardiovascular (CV) death in those at high risk for CV disease. Both Mediterranean diet groups receiving extra virgin olive oil or nuts had ~30% reduction in the composite outcome of CV events compared with the control diet (advice to reduce dietary fat) (22, 23). Furthermore, in the PREDIMED-Reus trial (the only centre where a yearly oral glucose tolerance test was part of the study protocol), diabetes incidence was significantly reduced by 52% when the two Mediterranean diet groups were pooled and compared to control (24). A recent SRMA of 3 RCTs and 38 prospective cohort studies found a beneficial effect of the Mediterranean diet on CV disease prevention in individuals with diabetes (25). This SRMA reported reductions in total CV and MI incidence in the pooled analysis of randomized controlled trials. In the pooled analysis of the prospective cohort studies which compared the highest versus lowest categories of Mediterranean diet adherence, an inverse association with total CVD mortality, coronary heart disease (CHD) incidence, CHD mortality, stroke incidence, stroke mortality and MI incidence was observed (25). Taken together, the Mediterranean dietary pattern can be a viable option for patients to transition onto after rapid weight loss using meal replacements.

### Portfolio Diet

The portfolio diet is a dietary portfolio of cholesterol-lowering foods, all of which have approved health claims by the U.S. Food and Drug Administration (FDA), Health Canada, and the European Association for the Study of Diabetes (EFSA). The four essential components of the Portfolio diet are viscous fiber (20g/d), soy protein (45g/d), plant sterols (2g/d), and nuts (45g/d). The first study evaluating the efficacy of the Portfolio diet was conducted in 2002 under metabolic feeding conditions in 46 hyperlipidemic adults (26). Participants randomized to the Portfolio diet group saw reductions in LDL-c of 28.6%, which was similar to the lovastatin group (30.9%), and significantly different from the control group (reductions of only 8%) over a 1-month period (26). In a longer follow-up study (N=356 hyperlipidemic patients) that simulated real-world conditions, intensive advice of the dietary portfolio (7 clinic visits in 6 months) and routine advice of the dietary portfolio (2 clinic visits in 6 months) resulted in significantly greater reductions in LDL-c (13.8% and 13.1%, respectively) when compared to the control group (reductions of 3%) that received advice on a low saturated fat diet (27). A SRMA of RCTs and non-RCTs of the portfolio dietary pattern found clinically meaningful reductions in the primary therapeutic lipid target for CVD prevention, LDL-C (~17% reduction), the established alternate lipid targets, non-HDL-C and apoB, as well as other established cardiometabolic risk factors, including triglycerides, blood pressure, and C-reactive protein, culminating in an improvement in estimated 10-year CHD risk (28). More recently, in a prospective cohort of 123,330 postmenopausal women in the United States, higher adherence to the Portfolio Diet was associated with a reduction in incident cardiovascular and coronary events, as well as heart failure (29). Overall, the Portfolio dietary pattern can also be a viable option for patients to transition onto after rapid weight loss using meal replacements.

## Future Directions

Despite a large body of evidence emphasizing the role of meal replacements in diabetes management, it is unclear whether these advantages translate to patients at risk for diabetes (i.e., prediabetes or metabolic syndrome). There is an urgent need to synthesize the evidence on liquid meal replacements and cardiometabolic risk factors in this at-risk population to understand the broader application of liquid meal replacements in clinical practice.

To address this gap, we are currently conducting a second SRMA of RCTs to evaluate the effect of liquid meal replacements as a temporary short to moderate term weight loss strategy in overweight or obese individuals with prediabetes and metabolic syndrome. We are following the same successful protocol used to conduct the SRMA of randomized controlled trials that investigated the effect of liquid meal replacements on established cardiometabolic risk factors in overweight and obese individuals with types 2 diabetes for the update of the EASD Clinical Practice Guidelines for nutrition therapy (ClinicalTirals.gov identifier, NCT02779790). Findings from this analysis are expected to be published in late-2022/early 2023.

## Conclusion

Incorporating meal replacements in comprehensive lifestyle interventions targeting 5-15% weight loss have shown important benefits in diabetes as shown by the Look AHEAD, DiRECT, and PREVIEW trials. Liquid meal replacements replacing 1-2 meals per day or all meals (total diet replacement) may have cardiometabolic advantages over conventional weight loss diets in overweight/obese adults with type 2 diabetes. Keeping in line with the new shift in clinical practice guidelines towards a focus on dietary patterns that provide greater choice, one potential dietary strategy can be to use meal replacements as a short-term strategy to induce weight loss prior to transitioning to a healthier dietary pattern such as, the Mediterranean or Portfolio dietary pattern for weight loss maintenance, sustained improvements in cardiometabolic risk factors, and associated reductions in cardiovascular disease. As adherence is the most important determinant for attaining the benefits of any diet, health professionals should advise patients on evidence-based dietary patterns (including meal replacements) that align best with their values, preferences, and treatment goals to achieve the greatest adherence over the long term.

## Data Availability Statement

The original contributions presented in the study are included in the article/supplementary material. Further inquiries can be directed to the corresponding author.

## Author Contributions

JN drafted the manuscript. CK and JS critically reviewed the manuscript for important intellectual content. All authors reviewed and approved the final manuscript.

## Funding

This work was supported by the Toronto 3D Knowledge Synthesis and Clinical Trials foundation.

## Conflict of Interest

JN has worked as a clinical research coordinator at INQUIS Clinical Research. He has also received research support from Glycemia Consulting Inc. CK has received grants or research support from the Advanced Food Materials Network, Agriculture and Agri-Foods Canada (AAFC), Almond Board of California, Barilla, Canadian Institutes of Health Research (CIHR), Canola Council of Canada, International Nut and Dried Fruit Council, International Tree Nut Council Research and Education Foundation, Loblaw Brands Ltd, the Peanut Institute, Pulse Canada and Unilever. He has received in-kind research support from the Almond Board of California, Barilla, California Walnut Commission, Kellogg Canada, Loblaw Companies, Nutrartis, Quaker (PepsiCo), the Peanut Institute,Primo, Unico, Unilever, WhiteWave Foods/Danone. He has received travel support and/or honoraria from the Barilla, California Walnut Commission, Canola Council of Canada, General Mills, International Nut and Dried Fruit Council, International Pasta Organization, Lantmannen, Loblaw Brands Ltd, Nutrition Foundation of Italy, Oldways Preservation Trust, Paramount Farms, the Peanut Institute, Pulse Canada, Sun-Maid, Tate & Lyle, Unilever and White Wave Foods/Danone. He has served on the scientific advisory board for the International Tree Nut Council, International Pasta Organization, McCormick Science Institute and Oldways Preservation Trust. He is a founding member of the International Carbohydrate Quality Consortium (ICQC), Executive Board Member of the Diabetes and Nutrition Study Group (DNSG) of the European Association for the Study of Diabetes (EASD), is on the Clinical Practice Guidelines Expert Committee for Nutrition Therapy of the EASD and is a Director of Glycemic Consulting and the Toronto 3D Knowledge Synthesis and Clinical Trials foundation. JS has received research support from the Canadian Foundation for Innovation, Ontario Research Fund, Province of Ontario Ministry of Research and Innovation and Science, Canadian Institutes of health Research (CIHR), Diabetes Canada, PSI Foundation, Banting and Best Diabetes Centre (BBDC), American Society for Nutrition (ASN), INC International Nut and Dried Fruit Council Foundation, National Honey Board (the U.S. Department of Agriculture [USDA] honey “Checkoff” program), Institute for the Advancement of Food and Nutrition Sciences (IAFNS; formerly ILSI North America), Pulse Canada, Quaker Oats Center of Excellence, The United Soybean Board (the USDA soy “Checkoff” program), The Tate and Lyle Nutritional Research Fund at the University of Toronto, The Glycemic Control and Cardiovascular Disease in Type 2 Diabetes Fund at the University of Toronto (a fund established by the Alberta Pulse Growers), The Plant Protein Fund at the University of Toronto (a fund which has received contributions from IFF), and The Nutrition Trialists Fund at the University of Toronto (a fund established by an inaugural donation from the Calorie Control Council). He has received food donations to support randomized controlled trials from the Almond Board of California, California Walnut Commission, Peanut Institute, Barilla, Unilever/Upfield, Unico/Primo, Loblaw Companies, Quaker, Kellogg Canada, WhiteWave Foods/Danone, Nutrartis, and Dairy Farmers of Canada. He has received travel support, speaker fees and/or honoraria from ASN, Danone, Dairy Farmers of Canada, FoodMinds LLC, International Sweeteners Association, Nestlé, Abbott, General Mills, Comité Européen des Fabricants de Sucre (CEFS), Nutrition Communications, International Food Information Council (IFIC), Calorie Control Council, and International Glutamate Technical Committee. He has or has had *ad hoc* consulting arrangements with Perkins Coie LLP, Tate & Lyle, and Inquis Clinical Research. He is a member of the European Fruit Juice Association Scientific Expert Panel and former member of the Soy Nutrition Institute (SNI) Scientific Advisory Committee. He is on the Clinical Practice Guidelines Expert Committees of Diabetes Canada, European Association for the study of Diabetes (EASD), Canadian Cardiovascular Society (CCS), and Obesity Canada/Canadian Association of Bariatric Physicians and Surgeons. He serves or has served as an unpaid scientific advisor for the Food, Nutrition, and Safety Program (FNSP) and the Technical Committee on Carbohydrates of IAFNS (formerly ILSI North America). He is a member of the International Carbohydrate Quality Consortium (ICQC), Executive Board Member of the Diabetes and Nutrition Study Group (DNSG) of the EASD, and Director of the Toronto 3D Knowledge Synthesis and Clinical Trials foundation. His spouse is an employee of AB InBev.

## Publisher’s Note

All claims expressed in this article are solely those of the authors and do not necessarily represent those of their affiliated organizations, or those of the publisher, the editors and the reviewers. Any product that may be evaluated in this article, or claim that may be made by its manufacturer, is not guaranteed or endorsed by the publisher.
